# Association Between Systemic Inflammation, Metabolic Syndrome and Quality of Life in Psoriasis Patients

**DOI:** 10.3390/life15020212

**Published:** 2025-01-31

**Authors:** Maria-Lorena Mustață, Carmen-Daniela Neagoe, Virginia-Maria Rădulescu, Ioana-Gabriela Dragne, Radu-Cristian Cîmpeanu, Lucrețiu Radu, Roxana-Viorela Ahrițculesei, Dragoș Forțofoiu, Maria-Cristina Predoi, Simona-Laura Ianoși

**Affiliations:** 1 Doctoral School, University of Medicine and Pharmacy of Craiova, 200349 Craiova, Romania; umlorena@yahoo.com (M.-L.M.); idragne@yahoo.com (I.-G.D.); cimpeanu_r@yahoo.com (R.-C.C.); roxana.blendea@gmail.com (R.-V.A.); fortofoiudragos@gmail.com (D.F.); 2 Department of Internal Medicine, Faculty of Medicine, University of Medicine and Pharmacy of Craiova, 200349 Craiova, Romania; dananeagoe2014@gmail.com; 3 Department of Medical Informatics and Biostatistics, Faculty of Medicine, University of Medicine and Pharmacy of Craiova, 200349 Craiova, Romania; 4 Department of Hygiene, Faculty of Medicine, University of Medicine and Pharmacy of Craiova, 200349 Craiova, Romania; lucretiu.radu@gmail.com; 5 Department of Morphology, Faculty of Medicine, University of Medicine and Pharmacy of Craiova, 200349 Craiova, Romania; predoi.cristina@yahoo.com; 6 Department of Dermatology, Faculty of Medicine, University of Medicine and Pharmacy of Craiova, 200349 Craiova, Romania; simonaianosi@hotmail.com

**Keywords:** psoriasis, systemic inflammation, metabolic syndrome, DLQI, PASI, leptin

## Abstract

Background/objectives: Psoriasis is a chronic inflammatory autoimmune disease with important systemic and psychosocial impacts. The association with metabolic syndrome (MS) impairs disease severity and negatively influences patient-reported outcomes, particularly their quality of life as measured by the Dermatology Life Quality Index (DLQI). This study aims to investigate the relationship between systemic inflammation, DLQI scores and disease severity, focusing on the persistent impact of MS on patient outcomes after one year of treatment. Methods: This retrospective cross-sectional study included 150 psoriasis patients, with 74 also meeting the diagnostic criteria for MS. Clinical and inflammatory markers such as systemic immune–inflammatory index (SII), cytokines (IL-17A, IL-23), leptin, BMI and triglycerides were analyzed alongside PASI and DLQI scores. Results: Patients with MS had significantly higher PASI and DLQI scores compared to those without MS, reflecting worse disease severity and quality of life (*p* < 0.01). Elevated SII levels were strongly associated with higher DLQI scores (*p* < 0.01). Despite considerable reductions in PASI scores over one year of treatment, DLQI scores indicated a persistent negative impact of MS on quality of life. Notably, markers of systemic inflammation, such as SII, leptin and cytokines, correlated positively with both PASI and DLQI scores, highlighting the role of systemic inflammation in disease burden. Conclusions: This study underlines the significant role of systemic inflammation and metabolic comorbidities in amplifying the burden of psoriasis. The persistent impact of MS on quality of life despite clinical improvement underscores the need for comprehensive treatment approaches targeting systemic inflammation, metabolic health and psychosocial factors to improve long-term outcomes.

## 1. Introduction

Psoriasis is an autoimmune disease clinically characterized by hyperkeratotic, elevated, clearly defined scaly plaques, typically located on the elbows, knees, scalp and sacral region [1,2,3]. The lesions can cause extreme pruritus, pain and visible scaling, leading to physical discomfort that causes stigma and limits social interaction and interpersonal interactions [4,5]. As a result, psoriasis patients frequently experience feelings of shame, anxiety or depression [6].

Numerous systemic diseases have been linked to psoriasis; approximately 75% of patients have been demonstrated to have at least one comorbid condition, and many have numerous comorbidities [7,8,9]. This association is a result of the chronic inflammatory changes and proinflammatory cytokines that are frequently elevated in psoriasis, which induce a continuous inflammatory state. According to the most recent research, the metabolic syndrome is one of the most prevalent comorbidities of psoriasis [10,11,12,13,14,15,16]. It is defined by the presence of at least three out of five criteria (abdominal obesity, hypertriglyceridemia, low HDL-cholesterol, hyperglycemia or diabetes mellitus, and hypertension) [17]. The presence of MS in psoriasis patients not only exacerbates clinical symptoms but also contributes to heightened systemic inflammation through mechanisms such as insulin resistance and endothelial dysfunction [18,19].

Psoriasis should be acknowledged as a complex systemic condition, transcending the simplistic classification of a skin condition, due to its significant influence on overall health, including a well-documented association with MS and its related physical and psychosocial complications [20]. Genetic predisposition, common inflammatory pathways and risk factors are mechanisms underlying the association between psoriasis and MS. Common genetic variants such as FUT2, UBE2L3, CDKAL1, SH2B3 and apolipoprotein E are more frequently found in psoriasis patients, having multiple functions that would explain their dual role in susceptibility to psoriasis and MS [21]. Moreover, common immunological mechanisms involving in particular Th and Th17 cell activation seem to link psoriasis to metabolic comorbidities [22]. The release of inflammatory mediators from psoriatic lesions, such as IL-1, IL-6, IL-17, TNF-a, IFN-a and IFN-y may have systemic effects that contribute to the atherogenesis process [22,23]. One finding that reinforces the relationship between the two conditions is the activity of TNF-a and IFN-y [23]. Extensive research has shown that the inflammatory state characteristic of psoriasis intensifies inflammation in the adipose tissue, which may trigger additional immune responses [24]. In psoriatic adipose tissue, T lymphocytes, dendritic cells, neutrophils, mast cells and macrophages, all of which are immune cells, play a key role in influencing cardiometabolic health and contribute to the development of obesity and insulin resistance. The release of adipokines such as chemerin, adiponectin, resistin, visfatin and C-reactive protein by macrophages and T-cells is the link between chronic systemic inflammation and obesity. Patients with psoriasis have elevated levels of these adipokines in the blood, which are thought to contribute significantly to the development of insulin resistance [25,26].

Nowadays, there is a varied range of therapeutic options for the management of psoriasis, which can be used topically or systemically. To date, there is no identified treatment that completely eradicates psoriasis. The primary objective is to achieve prolonged periods of remission. There have been significant improvements in the development of biologic treatments for psoriasis in the past years (Table 1). Molecules targeting TNF-a, IL-23 and IL-17 have demonstrated significant efficacy, which highlights the essential role of TNF-a and the IL-23/IL-17 axis in psoriasis development. Ongoing research is exploring novel therapies and alternative approaches, including natural products [27], JAK inhibitors, cell-based therapies and nanotechnology. Moreover, preclinical studies indicate that melatonin can reduce psoriasis skin lesion severity by normalizing inflammation and reducing oxidative stress [28].

Psoriasis patients’ quality of life is profoundly affected, from physical activities and interpersonal relationships to mental health. The psychological impact is amplified by social stigmatization, with a significant number of patients feeling excluded or judged because of the appearance of their skin [29,30,31]. A meta-analysis of 98 studies and 401,703 patients diagnosed with psoriasis found that they were at least 1.5 times more likely to experience depressive symptoms than those without the condition [32]. Similarly, female psoriasis patients were shown to be at greater risk to depression than their male counterparts, according to a recent systematic review [33].

An essential tool to highlight the degree of impairment and provide insight into the impact of the disease is the Dermatology Life Quality Index (DLQI). However, the DLQI does not appear to explore in detail certain psychological or economic aspects of the disease and is subjective in nature, as it is based on patients’ self-reported perception of the impact of their condition. Each patient may perceive his or her own condition differently, which may interfere with responses related to the discomfort created by the disease [34,35,36,37,38].

Along with the quality of life assessment, psoriasis severity is measured using the Psoriasis Area and Severity Index (PASI) score. In general, PASI and DLQI are directly related but the relationship between them is not always linear, in that patients with lower PASI may continue to report a high DLQI even after clinical symptoms have improved, reflecting the persistence of the psychosocial impact of the disease [39,40,41].

Following the latest research, the systemic immune–inflammatory index (SII), along with C-reactive protein, ferritin and ESR, has emerged as a valuable new inflammatory biomarker that has recently been introduced to assess the association between chronic inflammatory state and multiple chronic diseases, including various types of neoplasms, metabolic disorders and psoriasis [42].

Previous studies have extensively investigated the relationship between the clinical severity of psoriasis and quality of life [43,44]. Nonetheless, the combined impact of the metabolic syndrome and the role of inflammation on the quality of life of psoriasis patients has yet to be investigated.

This study aims to explore the relationship between DLQI, PASI and associated comorbidities, particularly metabolic syndrome, in patients with psoriasis. In our research, we analyzed how clinical and inflammatory aspects, such as BMI, triglycerides, glycemia, leptin, SII and the cytokines IL-17A and IL-23, influence quality of life as measured by DLQI, in psoriasis patients after one year of treatment.

## 2. Materials and Methods

### 2.1. Patient Selection

This retrospective cross-sectional study initially included a cohort of 189 patients diagnosed with psoriasis vulgaris. Following the inclusion and exclusion criteria, 150 patients were enrolled in the study, consisting of two groups of patients, 76 of them diagnosed with psoriasis and 74 with both psoriasis and metabolic syndrome. The study ran for one year, from January 2022 to January 2023. The 150 patients included in the study were monitored the entire year, with assessments performed at 3 months, 6 months and at the end of the year. The patients were enrolled consecutively as they presented for psoriasis management. All patients received written informed consent. The study received the approval of the Ethics Committee of the University of Medicine and Pharmacy of Craiova (approval number 195/20 September 2022) and adhered to the principles described in the Declaration of Helsinki (2004).

Inclusion criteria were patients older than 18 years of age with a clinical diagnosis of psoriasis, regardless of its severity.

Exclusion criteria were as follows:

- Patients with other concomitant autoimmune diseases (2 patients with rheumatoid arthritis and 1 patient with systemic lupus erythematosus);
- End-stage renal disease: 3 patients;
- Advanced liver cirrhosis: 4 patients;
- Recent myocardial infarction (<1 year): 2 patients;
- Active malignancies: 9 patients;
- Patients diagnosed with asthma: 6 patients;
- Patients with a history of alcohol or drug use (which may alter metabolic or inflammatory markers): 12 patients.

### 2.2. Laboratory Investigations

Laboratory investigations were collected from venous blood from the antecubital vein of each patient: complete blood count, complete lipid profile (total cholesterol, LDL-cholesterol, HDL-cholesterol, triglycerides), transaminases (ALAT, ASAT), blood glucose and markers of inflammation: C-reactive protein (CRP), leptin, IL-17 and IL-23. Systemic immune–inflammation (SII) index was calculated using the formula: absolute neutrophil count X (total platelet count/absolute lymphocyte count) ratio. Metabolic syndrome was diagnosed according to the National Cholesterol Education Program-Adult Treatment Panel (NCEP-ATP III) criteria using at least three of the five components: abdominal circumference (AC) ≥ 88 cm in women and ≥102 cm in men; serum triglyceride (TG) value ≥ 150 mg/dL (or on lipid-lowering treatment); HDL-cholesterol < 50 mg/dL in women and <40 mg/dL in men, or on cholesterol-lowering treatment; systolic blood pressure (BP) ≥ 130 mmHg and/or diastolic BP ≥ 85 mmHg or on antihypertensive treatment; fasting blood glucose ≥ 100 mg/dL, or undergoing blood glucose-lowering treatment [16].

### 2.3. Assessment of Psoriasis Disease Severity

Assessment of psoriasis severity was performed using the Psoriasis Area and Severity Index (PASI) score, which combines manifestations related to disease severity, such as erythema, induration and desquamation, with the size of the affected area, measured as a percentage. Each region was assigned a score that highlighted the degree of involvement of that region, and a score that recorded the severity of psoriasis. The PASI ranges from 0 to 72, with four areas of the body being assessed: head and neck, upper limbs, trunk and lower limbs, and in each area, the severity index is assessed by signs such as erythema, degree of induration and flaking. Each of these signs is rated on a scale of 1 to 5. Thus, according to the PASI score, psoriasis is categorized into three severity grades: below 10 signifies mild psoriasis, between 10 and 20, moderate psoriasis and above 20, severe psoriasis [39].

Patients’ quality of life was estimated using the DLQI questionnaire which explores some relevant dimensions of the patient’s life that may be affected by the disease. The questionnaire comprises 10 questions that assess patients’ perceptions of the impact of psoriasis on their quality of life and includes items such as symptoms, emotions or feelings, daily activities, leisure and sport, work or study, social contact and treatment. Based on their performance in the week prior to completing the questionnaire, patients answer these questions, giving scores ranging from 0 to 3: 0 means not at all; 1—mild; 2—severe; and 3—very severe. The total DLQI score is 30, with a higher score representing a more impaired quality of life [35].

### 2.4. Statistical Analysis

Statistical analyses were performed using the SPSS (Statistical Package for Social Sciences) software, specifically version 26 developed by SPSS Inc. in Chicago, IL, USA. In this study, continuous variables were characterized by their mean and standard deviation (SD), providing a clear summary of the data’s central tendency and variability. For categorical and ordinal variables, we utilized frequency distributions and percentages to offer a comprehensive overview of the different categories present in the data.

To evaluate the distribution of the data, we applied the Kolmogorov–Smirnov/Shapiro–Wilk tests, which assess the normality of the dataset. This step was crucial in determining the appropriate statistical tests for further analysis.

For comparing the variables between the study group and the control group, we adopted different statistical methods based on the nature of the data. For data that met the assumptions of normal distribution, we employed the independent *t*-test. In contrast, when dealing with continuous variables that did not follow a normal distribution, we utilized non-parametric tests, specifically Kendall’s tau-b, Mann–Whitney U and Kruskal–Wallis tests. Additionally, for categorical data, we implemented the chi-square test to examine the relationships between variables.

To conclude our analyses, we considered a *p*-value of less than 0.05 to be statistically significant, indicating a meaningful difference or correlation in our study results. This threshold helped ensure the robustness of our findings.

## 3. Results

Following the application of the established inclusion and exclusion criteria, the study group was composed of 150 patients. This cohort included 58 females and 92 males, with ages ranging from 26 to 76 years. The mean age of the participants was 53.8 ± 11.7 years, indicating a broad age distribution among the subjects.

Analysis of the gender distribution revealed that male patients constituted 61.3% of the group, with a mean age of 55.3 ± 12.1 years. In comparison, female patients represented 38.7% of the cohort, with a mean age of 51.5 ± 10.6.

In our study, the prevalence of comorbidities among patients is striking: 86.7% are either obese (56%) or overweight (30.7%), with only a small fraction, 13.3%, falling into the normal weight category. Hypertension also plays a critical role in this context, as 52% of the 150 patients reported having high blood pressure, highlighting a significant public health concern.

Regarding treatment, only 13.3% of the patients were prescribed Methotrexate, while 86.7% received biologics. The distribution of biological therapies reflects a diverse approach: 21.3% were treated with anti-IL-17, 30.7% with anti-IL-23 and 34.7% with anti-TNF-a therapies.

The analysis involved monitoring various parameters, including triglycerides, blood glucose, HDL cholesterol, blood pressure and inflammatory markers such as leptin, IL-17A and IL-23, with a focus on the differences between groups and the relationships among the variables.

The mean BMI value within the study population was found to be 3.43 ± 0.717, suggesting a relatively uniform distribution among the participants. The median value of 4 further corroborates that most individuals fall within a distinct BMI category. Baseline triglyceride levels exhibited a high mean of 209.15 mg/dL, accompanied by considerable variability (standard deviation of 127.67 mg/dL). The median triglyceride level of 185.5 mg/dL indicates that while most subjects present elevated values, these do not reach extreme levels. Conversely, HDL-cholesterol at baseline demonstrated a mean of 94.16 mg/dL and a median of 91.29 mg/dL, reflecting lower variability relative to triglycerides.

In addition, inflammatory markers such as leptin and IL-17-A were observed at notably high levels, with means of 538.96 and 593.24, respectively; these findings suggest significant inflammation among the patient population. This aspect is particularly pertinent in examining the interactions among inflammation, BMI and quality of life.

The analysis of variance (ANOVA) revealed significant differences across groups with respect to most variables. For instance, baseline triglyceride levels yielded a *p*-value of 0.01, indicating clear discrepancies between the BMI categories. Blood glucose levels also demonstrated significance, with a *p*-value < 0.01, highlighting the influence of body mass on glucose metabolism. Blood pressure measurements, both at baseline and after one year, presented *p*-values < 0.01, confirming a substantial correlation between BMI and blood pressure. In contrast, *p*-value of 0.128 for HDL cholesterol after one year suggests the absence of significant differences among groups, potentially reflecting the stability of this parameter over time. A *p*-value of 0.065 for the inflammatory marker IL-23 indicates a trend toward statistical significance, although it does not meet the established threshold.

The strength of the relationships among variables was evaluated using the coefficient of determination (R-squared). For instance, the R-squared value for baseline triglycerides concerning BMI group is 0.191, indicating a moderate association. Conversely, the association between leptin and DLQI is weaker, with an R-squared value of 0.091.

All participants had previously received the diagnosis of psoriasis. Significantly, approximately half of these patients—specifically 74 individuals, or 49.3%—also presented with metabolic syndrome (MS). Consequently, the study group was bifurcated into two distinct subgroups: the PSO group, which consisted of patients diagnosed solely with psoriasis, and the PSO–MS group, which included those diagnosed with both psoriasis and metabolic syndrome.

### 3.1. Analysis of Study Subgroups: PSO Versus PSO–MS

A Mann–Whitney U test was run to determine if there were differences in the ages of patients from both subgroups. Distributions of the ages for PSO and PSO–MS patients were similar, as assessed by visual inspection. The mean age for PSO patients (51.34 ± 12.54) was smaller than the mean age for PSO–MS patients (56 ± 10.29), but the difference was not statistically significantly different, U = 2628.0, z = −0.69, *p* = 0.48.

The analysis revealed a statistically significant difference in gender distribution between the two subgroups. In the subgroup of patients without metabolic syndrome, gender representation is relatively comparable; however, the subgroup of patients with metabolic syndrome displays a notable contrast. Specifically, within the PSO–MS subgroup, females constitute 27.0% of the patients, while males account for 72.9% of the PSO subgroup (χ^2^(1) = 8.3, *p* < 0.01). Furthermore, it is noteworthy that all patients in the PSO–MS subgroup have hypertension, in comparison to only 5.2% of patients in the PSO group (χ^2^(1) = 134.81, *p* < 0.01).

The patients in the PSO subgroup exhibited a median weight of 82.6 kg, which was significantly lower than that of the patients in the PSO–MS subgroup, who demonstrated a median weight of 100.7 kg (*p* < 0.01). Additionally, the abdominal circumference (AC) was notably greater in patients with metabolic syndrome, recording a median value of 109.5 cm, compared to the PSO patients, whose median AC was 91.47 cm (*p* < 0.01). Similar patterns were observed in the analysis of body mass index (BMI); patients in the PSO subgroup without metabolic syndrome had a median BMI of 28.4, significantly lower than the median BMI of 32.7 in patients within the PSO–MS subgroup (*p* < 0.01). These patient characteristics are detailed in Table 2.

In patients diagnosed with PSO–MS, there was a notable elevation in the levels of cholesterol, triglycerides and glycemia when compared to individuals with PSO alone. The observed differences in these biochemical parameters were statistically significant, with a *p*-value < 0.01, as illustrated in Table 2. This suggests a potential link between the presence of metabolic syndrome and the disturbance of lipid and glucose metabolism in patients with psoriasis. On the other hand, patients within the PSO–MS subgroup exhibited a reduced concentration of high-density lipoprotein (HDL), which is often regarded as the “good” cholesterol and is crucial for cardiovascular health.

**Figure 1:**
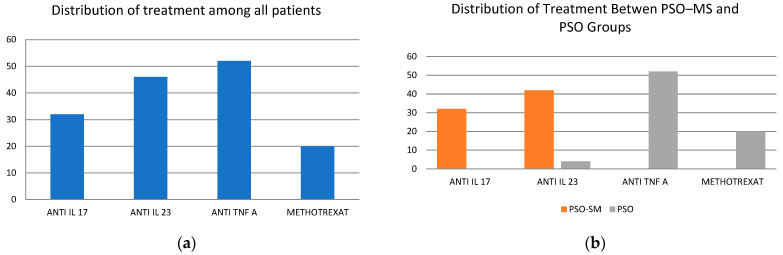
Treatment distribution: (**a**) Distribution of treatment among all patients; (**b**) Distribution of treatment between PSO–MS and PSO groups.

Leptin levels were significantly elevated in patients classified within the PSO–MS subgroup in comparison to those with psoriasis (PSO), with a statistically significant difference observed (*p* < 0.01). A comparative analysis of interleukins IL-17A and IL-23 revealed noteworthy findings; specifically, the PSO–MS group demonstrated higher concentrations of both interleukins. IL-17A exhibited a statistically significant difference relative to the PSO group (*p* < 0.01). In contrast, the levels of IL-23 were found to be comparable between the two groups. A summary of the treatments administered by patient group is presented in Figure 1.

A comparative analysis of patients with and without MS after treatment highlighted significant improvements in patients’ quality of life and metabolic parameters. The treatment had a distinct impact on each group, and the analysis of the results reveals promising advancements and areas that warrant further investigation.

When it comes to quality of life, patients with MS experienced a more pronounced improvement compared to those without MS. In the group treated with anti-IL-23, the median DLQI score remained steady at 4, but the DLQI grade decreased from 5 to 4, indicating a moderate improvement. Conversely, patients treated with anti-IL-17 showed a more significant improvement, with median DLQI decreasing from 4 to 3.5 and DLQI class decreasing from 5 to 3.5. This suggests that treatment with anti-IL-17 had a more positive impact on quality of life, offering hope for patients with MS.

The changes were less evident in patients without MS. For those treated with methotrexate, the median DLQI increased from 3 to 5, indicating a possible deterioration in quality of life. For treatment with anti-TNF-a, the median remained stable at 3.5, and the DLQI class did not change. The group treated with anti-IL-23 showed a slight increase in median DLQI from 3.5 to 4.5.

One of the most important metabolic markers analyzed was triglyceride levels. In MS patients, treatment led to significant decreases. In the group treated with anti-IL-23, the mean triglyceride level decreased from 316.5 ± 165.7 mg/dL to 192.3 ± 63.8 mg/dL. Similarly, in patients treated with anti-IL-17, the triglyceride level decreased from 236.3 ± 59.0 mg/dL to 165.9 ± 52.5 mg/dL. These reductions, which are indicative of improved lipid profiles, reflect a significant improvement in the metabolic health of MS patients.

In patients without MS, the decreases were less marked but evident. In the methotrexate-treated group, triglyceride levels decreased from 126.9 ± 31.9 mg/dL to 101.2 ± 24.6 mg/dL. Treatment with anti-TNF-a resulted in a decrease from 144.9 ± 73.5 mg/dL to 109.3 ± 50.7 mg/dL. For anti-IL-23, triglycerides remained relatively stable, decreasing slightly from 111.0 ± 31.0 mg/dL to 109.5 ± 31.7 mg/dL.

Mean glycaemia did not change significantly in either group. In patients with MS, glycemic values remained stable for anti-IL-23 (163.8 ± 68.7 mg/dL) and anti-IL-17 (133.6 ± 34.5 mg/dL). Also, in patients without MS, glycemic values were constant: 98.3 ± 14.0 mg/dL for methotrexate, 93.0 ± 18.2 mg/dL for anti-TNF-a and 88.8 ± 2.1 mg/dL for anti-IL-23. This stability in glycemic values suggests that the treatments did not have a significant impact on blood sugar levels.

HDL, an indicator of cardiovascular health, significantly improved MS patients. For anti-IL-23 treatment, the mean HDL level increased from 37.0 ± 6.7 mg/dL to 51.3 ± 5.6 mg/dL. For anti-IL-17, the increase was similar, from 42.0 ± 10.5 mg/dL to 52.6 ± 8.3 mg/dL. These results indicate an improvement in the lipid profile of MS patients.

Patients without MS maintained healthier HDL levels throughout the study. In the methotrexate-treated group, HDL increased from 56.4 ± 8.7 mg/dL to 58.2 ± 9.4 mg/dL. Regarding anti-TNF-a, the increase was from 60.6 ± 15.2 mg/dL to 62.8 ± 13.4 mg/dL, and for anti-IL-23, from 54.7 ± 4.2 mg/dL to 60.5 ± 6.4 mg/dL.

### 3.2. PASI Values Analysis

For all patients, the PASI values were measured before the initiation of PSO treatment. A Mann–Whitney U test was run to determine if there were differences in PASI values between males and females. Distributions of PASI values for males and females were similar, as assessed by visual inspection. The median PASI for males (21.4) was higher than for females (18.8), yet the difference was not statistically significantly different, U = 3036.0, z = 1.42, *p* = 0.15. A similar test was run to determine whether there were differences in PASI values between patients with and without MS. The analysis of PASI values revealed notable differences between the two patient categories. Specifically, the PSO–MS subgroup demonstrated a higher median PASI value of 22.3, in contrast to the PSO subgroup, which had a median value of 18.4. This difference was found to be statistically significant, as indicated by a U = 3376.0, z = 2.12 and a *p*-value of 0.03.

A Kendall’s tau-b correlation analysis was conducted to examine the relationship between PASI values and the duration between the onset of psoriasis and the commencement of therapy, measured in months. The findings indicated a moderate positive association, with statistical significance reported as τb = 0.25, *p* < 0.01. This suggests that a longer interval between the diagnosis of PSO and the initiation of treatment correlates with higher PASI values.

The analyses of the patients participating in this study were conducted regularly, with the PASI data recorded at three months, six months and one year.

The results showed that as patients received treatment, the mean PASI score decreased significantly. Specifically, the median PASI scores were as follows: at the initiation of treatment, the median was 20.3; at three months, it was 7.07; at six months, it was 3.67; and at twelve months, it was 2.10.

We applied the Kruskal–Wallis test to assess the differences among the four subgroups of patients categorized by their PASI scores. The results indicated statistically significant differences between these subgroups at various time points, with *p*-values as follows: PASI at initiation: 0.03; PASI at three months: 0.01; PASI at six months: <0.01; and PASI at twelve months: <0.01.

The analysis of the parameters IL-17A, IL-23, baseline leptin levels and leptin levels at the 12-month mark was conducted, taking into account two patient subgroups (PSO and PSO–MS) as well as the PASI scores recorded during the specified time periods. The findings are succinctly presented in Table 3.

Furthermore, Kendall’s tau-b correlations were performed to evaluate the relationship between PASI levels and the clinical parameters detailed in Table 2, encompassing a sample size of 150 participants. The analysis unveiled primarily weak-to-moderate positive associations between the initial PASI index at the commencement of PSO treatment and the parameters listed in Table 2. Notably, most of the tests yielded statistically significant results, as presented in Table 4.

Our research has unveiled significant positive correlations between weight (kg) and PASI values, with a robust Kendall’s tau-b of τb = 0.19 and a highly significant *p*-value of *p* < 0.01. Similarly, BMI shows a positive correlation with PASI values, with a substantial τb = 0.18 and a *p*-value of *p* < 0.01. These findings have direct implications for the management of dermatological and metabolic health conditions. Additionally, we identified correlations for cholesterol, triglycerides (TG), glycaemia, leptin and IL-23, providing a comprehensive understanding of the factors influencing PASI values. Furthermore, lower HDL values were associated with higher PASI values, with a noteworthy τb = −0.13 and a *p*-value of *p* < 0.01. All other parameters were not correlated to PASI values (*p* > 0.05) (Table 4).

### 3.3. Dermatology Life Quality Index (DLQI) Analysis

Our database confidently includes 150 patients, all classified according to their Dermatology Life Quality Index (DLQI) scores. Of these patients, 9.3% fell into DLQI class 3, 54.7% into DLQI class 4 and 36% into DLQI class 5, with no patients represented in DLQI classes 1 or 2. An analysis of the data by the various DLQI classes indicates the following median scores: patients classified in class 3 exhibited a median DLQI score of 10, those in class 4 demonstrated a median score of 14.63 and patients in class 5 recorded a median score of 24.3.

Statistical analysis revealed significant differences among the DLQI groups, particularly within the PSO and PSO–MS subgroups. These compelling results were obtained through the Mann–Whitney statistical test, yielding U = 1192.0, z = −6.11 and *p* < 0.01. The *p*-value, less than 0.01 in this case, indicates that the observed differences among the DLQI groups are statistically significant and not due to random variation.

**Figure 2:**
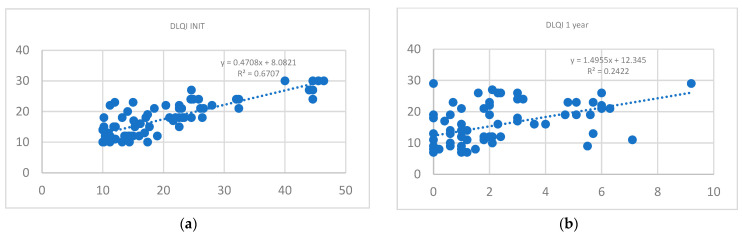
DLQI correlation: (**a**) correlation for initial DLQI; (**b**) correlation for final DLQI.

A correlation analysis of the DLQI values at the commencement and conclusion of the study indicated significant positive correlations for initial values (0.68, *p* < 0.01) between the categories, as presented in Figure 2.

An evaluation of the quality of life among the study participants revealed that cases were exclusively distributed across DLQI classes 3, 4 and 5, as we said before. A significant majority of the patients, regardless of gender, were classified within DLQI class 4, accounting for 54.7% of the sample. Additionally, 36.0% of patients were categorized in DLQI class 5, while only 9.3% were classified in class 3, as illustrated in Table 5. The statistical analysis conducted to assess the distribution by DLQI class with gender yielded χ^2^(2) = 2.19, *p* = 0.33, indicating an absence of statistically significant differences among the categories. Therefore, it can be concluded that gender does not influence the quality of life within the context of this study.

Kendall’s tau-b correlations were performed to evaluate the relationship between DLQI and the clinical parameters presented in Table 2 for all 150 participants. The analysis showed weak to moderate associations between the initial DLQI scores at the beginning of psoriasis treatment and the parameters listed in Table 2. Most tests yielded statistically significant results, which are detailed in Table 6.

Moreover, data from the entire group were analyzed to determine the correlation between DLQI and the initial PASI score. A moderate positive correlation was found between these two variables, with a τb value of 0.56 and a *p*-value < 0.01. Additionally, the correlation between DLQI and PASI values at the one-year mark was also examined. It revealed a weak correlation; however, there were statistically significant differences between the groups, as indicated by a *p*-value < 0.01.

The data collected from patients regarding DLQI scores, PASI scores and the PSO and PSO-SM subgroups were further analyzed using the Kruskal–Wallis test. The results indicated statistically significant differences among all categories, as shown by the *p*-values presented in Table 7.

Analyzing the distribution of cases according to SII in relation to their belonging to DLQI classes, it was observed that 54.7% of cases were in the 4th DLQI class, 36.0% were cases in the 5th DLQI class and only 9.3% were cases in the 3rd DLQI class (Table 8).

It can be seen that the cases in class 3 DLQI were predominantly cases with low SII (8%) and those with high SII were only 1.3%. The situation changes as the DLQI increases. If for DLQI 4 the distribution of cases suffers a difference of about 2% of the cases, at DLQI 5 the cases with high SII are twice as many as those with SII below the risk value.

Furthermore, from a statistical point of view, it was found that there are significant differences between the categories of SII cases relative to the DLQI classes, obtaining a *p*-value < 0.01 when applying the Chi-square test. A moderate positive correlation was also found between categories (*p* = 0.32).

In addition to our previous analyses, we employed a general linear model to evaluate the impact of the DLQI on various health indicators at the study’s commencement and conclusion. Our results included multivariate and univariate tests, post hoc analyses and R-squared values to clarify the relationships among health indicators.

We specifically examined how the DLQI index related to metabolic syndrome indicators, triglyceride levels and leptin concentrations. Our analysis featured two categorical predictors:

- DLQI at the beginning of the study (Level 3: 14 observations, Level 4: 82 observations, Level 5: 54 observations).
- DLQI at one year (Level 3: 40 observations, Level 4: 68 observations, Level 5: 42 observations).

The dependent variables included metabolic syndrome (MS), triglycerides (TG) and leptin (LEPTIN) values measured after one year.

Multivariate analyses were conducted utilizing Pillai’s Trace, Wilks’ Lambda, Hotelling’s Trace and Roy’s Largest Root to evaluate the combined effects of predictors on dependent variables. Subsequently, univariate tests were performed to identify specific impacts, with post hoc analyses employing Bonferroni adjustments to examine pairwise differences between the predictors’ levels. R-squared values were calculated to assess the proportion of variance explained by the model for each dependent variable.

The analyses revealed significant effects for both the predictors and their interaction. Notably, initial DLQI values exhibited a considerable overall effect on the dependent variables, as indicated by Wilks’ Lambda (*p* < 0.01). Similarly, DLQI values after one year demonstrated a highly significant influence (Wilks’ Lambda, *p* < 0.01). The interaction term (initial DLQI * DLQI after one year) also exhibited significance (*p* < 0.01), suggesting a dependency of the DLQI impact on its final level.

In terms of univariate test results, significant main effects were observed for metabolic syndrome concerning both initial DLQI (F = 8.133, *p* < 0.01) and DLQI after one year (F = 23.365, *p* < 0.01). In contrast, the interaction term did not yield significance (*p* = 0.364). For triglyceride levels, both predictors showed significant effects with initial DLQI values yielding F = 8.055 (*p* < 0.01) and DLQI values after one year resulting in F = 10.026 (*p* < 0.01). Furthermore, leptin levels displayed significant effects as well, with initial DLQI (F = 5.876, *p* = 0.004) and DLQI after one year (F = 10.154, *p* < 0.01) both proving significant. Conversely, HDL values demonstrated non-significant results across all predictors (*p* > 0.05).

The post hoc analyses identified significant pairwise differences in metabolic syndrome, particularly between levels 3 and 4, 3 and 5, and 4 and 5 (all *p* < 0.01). These findings imply that higher initial DLQI values are associated with increased severity of MS. Additionally, notable differences were observed in baseline triglyceride levels across all initial DLQI values (*p* < 0.01), indicating a rise in triglyceride levels corresponding with higher initial DLQI. Leptin concentrations also significantly increased with ascending initial DLQI value levels.

## 4. Discussion

A wide variety of studies characterize the impact of psoriasis on quality of life, most of them reinforcing the perception that the psycho-emotional impact of psoriasis is as important as its physical consequences, contributing equally to the overall morbidity of the disease [45,46,47,48]. The indicators of severity of psoriasis are clinical manifestations and the total body surface area affected, but the assessment of clinical severity can sometimes be difficult because physical symptoms do not always indicate the clinical severity of the disease [40,41,49]. Although some patients may have a fairly small involved body surface area, the location and appearance of the lesions can be a burden on participation in daily activities, weighing heavily on emotions and self-image. For example, a patient whose psoriasis is classified as mild psoriasis, with lesions located in an easily visible area (such as on the hands or elbows), is likely to be more socially and psychologically affected than a patient with the same lesions, but located in a less visible area (for example, on the abdomen) [50,51].

Psoriasis is a complex, immune-mediated skin condition that manifests clinically as a consequence of a complex interaction between the nervous, immune and cutaneous systems. This interaction is referred to as the nervous–skin–immune system chain or the neuro-immune-cutaneous system which is characterized by a close collaboration between keratinocytes, neuropeptide mediators and immune system cells, and its activation contributes to a state of continuous inflammation [52,53]. Psychological stress is a recognized trigger and aggravating factor in psoriasis, primarily due to the activation of the hypothalamic–pituitary–adrenal (HPA) axis, which generates the release of cortisol and catecholamines that can have proinflammatory effects by dysregulating cortisol function in the skin [54,55,56,57].

Psoriasis is mediated by T lymphocytes, in particular Th1 and Th17, and dendritic cells that are activated and elevated in skin lesions and migrate and release proinflammatory cytokines such as IL-1 and IL-6, but also TNF-a, which favor keratinocyte proliferation and inflammation. Several studies have reported elevated levels of proinflammatory cytokines, CRP and TNF-a among patients suffering from major depressive disorders [58].

The results of this study underscore the profound impact of psoriasis on patients’ quality of life, especially in the presence of metabolic comorbidities. In order to manage psoriasis efficiently, an in-depth understanding of its complex nature and the comorbidities present is imperative. The aim of this research was to better understand how the metabolic syndrome affects the quality of life of psoriasis patients. Comparative analysis between the PSO and PSO–MS groups revealed significant differences in both disease severity as expressed by PASI and quality of life as evidenced by DLQI.

The differences between the PSO and PSO–MS groups highlight the combined burden of metabolic syndrome among psoriasis patients. The components of the metabolic syndrome, such as obesity and dyslipidemia, are not only diagnostic markers, but actively contribute to systemic inflammation and disease severity [59,60].

The proinflammatory status that metabolic syndrome promotes, given its pathophysiology, exacerbates the severity of psoriasis. Psoriasis and metabolic syndrome both exhibit chronic systemic inflammation, which is a distinguishing feature of both conditions [61,62]. Its components, which include obesity, hypertension, dyslipidemia and insulin resistance, exacerbate cardiovascular risk, complicate disease management and impact the quality of life of patients [63]. In comparison to individuals without metabolic syndrome, patients with both psoriasis and metabolic syndrome are at an increased risk of cardiovascular events, including stroke and myocardial infarction, according to numerous studies [60,61,62,63,64]. The metabolic abnormalities in MS, when combined with the systemic inflammation present in psoriasis, synergistically increase the cardiovascular risk in these patients. This is accompanied by a lower quality of life, which is translated into poor physical and mental health [65].

Patients in the PSO–MS group, as expected, had a higher degree of hypertension than those in the PSO group, where only 5.26% of them had hypertension (*p* < 0.01). Endothelial dysfunction is the main mechanism linking chronic inflammation in metabolic syndrome and psoriasis to cardiovascular risk [63]. Due to proinflammatory cytokines, such as IL-17, IL-23 and TNF-a existent in patients with psoriasis and MS, endothelial cell function is destroyed [66]. The bioavailability of nitric oxide, an essential molecule produced by endothelial cells which facilitates vasodilatation, is disturbed, thereby increasing vascular resistance and developing arterial stiffness. Chronic inflammation contributes to changes in the structure of the arterial walls, including collagen deposition and loss of elasticity, thus contributing to arterial hypertension [67,68].

In addition, oxidative stress exacerbates endothelial dysfunction. Common in MS due to obesity and dyslipidemia, oxidative stress produces reactive oxygen species (ROS) that degrade nitric oxide and facilitate the formation of peroxynitrite, a toxic compound that damages endothelial cells [68,69]. Inflammation and lipid abnormalities promote endothelial activation which is characterized by an increase in the adhesion molecules ICAM-1 and VCAM-1, which facilitates the recruitment of monocytes to the endothelium, initiating and accelerating the development of atherosclerosis [70].

In line with previous findings, the lipid profile and weight were more elevated in patients with PSO–MS, which is in concordance with the defining features of MS. These findings align with the diagnostic criteria and emphasize its role in exacerbating the inflammatory and metabolic dysregulations observed in our study.

Results also indicate a significant gender disparity, with a higher prevalence of MS among male patients. These findings are supported by previous research linking MS to sex-specific inflammatory and metabolic responses. A cross-sectional study of 44,715 patients using The Health Improvement Network (THIN) database showed a higher prevalence of male patients among those with psoriasis and metabolic syndrome [71]. Moreover, the positive correlation between weight and PASI (*p* < 0.01) highlights the role of obesity as a significant factor influencing the severity of psoriasis. Obesity contributes to a pro-inflammatory state that exacerbates psoriasis by secreting adipokines, such as leptin and resistin, which stimulate Th17 and Th1 cells, thereby increasing Il-17 and TNF-a cytokines, key factors involved in the pathogenesis of psoriasis [72,73]. In addition, elevated levels of cholesterol, triglycerides and glycemia were also positively correlated with PASI score, emphasizing the impact of dyslipidemia and metabolic syndrome on the severity of psoriasis [74].

A notable clinical implication of this study is the persistent impact of MS on patient-reported outcomes, as measured by DLQI scores, even after one year of treatment. In terms of PASI scores, at baseline, patients in the PSO–MS group had higher values than those in the PSO group, reflecting the greater severity of disease in the presence of metabolic syndrome. Over one year of treatment, PASI decreased in both subgroups, but the PSO–MS patients maintained higher scores in each of the analyzed intervals (3, 6 and 12 months), emphasizing that MS not only worsens systemic inflammation, but also slows the response to treatment, as has been shown in other studies [75,76]. For instance, a study on brodalumab showed that obese patients (BMI ≥ 30) had a significantly lower proportion in achieving PASI 100 at week 24 (53.21%) compared to overweight patients (67.06%) [77]. Similarly, in a cohort study on risankizumab, patients with cardiometabolic diseases (CMD) showed reduced long-term responses, mostly at two years after the beginning of the treatment, with PASI 100 achieved by 65.4% of patients without CMD, compared to 52.4% of patients with CMD [78]. On the other hand, DLQI scores indicated persistent impairment despite improvement in PASI. Although PASI had significant decreases over time, patients in the PSO–MS group reported higher DLQI scores compared with the PSO subgroup, suggesting exacerbation of the psychosocial burden of psoriasis complicated by MS, most likely due to persistent systemic inflammation and its effects on patients’ perception of their condition. Even after one year of treatment, DLQI remains statistically significant in the PSO–MS group. This highlights the disconnect between clinical improvement and quality of life as perceived by patients, particularly those with various associated comorbidities, especially metabolic syndrome.

The DLQI scores were exclusively distributed in classes 3 to 5, suggesting moderate to severe impairment; more than half of the patients (54.7%) were part of class 4, while 36% were in class 5, highlighting the substantial impact of the disease even after treatment. These particular results from our study highlight the need for further research exploring the clinical, psychosocial and metabolic factors that shape the interaction between PASI and DLQI. The majority of existing studies focus on the relationship between PASI and DLQI in isolation, without exploring in depth the factors that contribute to the incongruence between these scores [79]. There is a lack of longitudinal studies that follow the evolution between PASI and DLQI over the long term in patients with psoriasis and metabolic comorbidities, and that examine whether changes in clinical severity, regarding the clinical improvement of psoriasis, might lead to proportional improvements in quality of life.

A recent study [40], which included an analysis of data from four phase-3 clinical trials, investigated the relationship between changes in PASI and DLQI scores in patients with moderate to severe psoriasis and showed that reduction in psoriasis severity was associated with significant improvements in patients’ quality of life, as reflected by DLQI scores. In our study, patients with associated metabolic syndrome, although they had an improvement in psoriasis as evidenced by their PASI score, their quality of life was poorer compared to patients in the PSO group without other comorbidities, reflecting the psychosocial impact that metabolic syndrome exerts on patients. Although there is a limited amount of data in this area, most indicate a correlation between MS and a decrease in quality of life, particularly in patients who also suffer from depression [80,81,82].

Metabolic syndrome amplifies the negative perception of patients’ health. The symptoms associated with metabolic syndrome, in particular the high risk of cardiovascular disease, contribute to a heightened sense of vulnerability, with patients often perceiving these signs as indicators of a general deterioration in health [83].

Aesthetic factors and social pressure also play an essential role. Abdominal obesity, a common component of the metabolic syndrome, can in itself attract social stigmatization, which can amplify feelings of inadequacy and devaluation, contributing to low self-confidence [84].

Moreover, correlation analyses between DLQI and metabolic parameters, including BMI, triglycerides and glycemia, reinforce the role of MS as a factor in reducing the quality of life among psoriasis patients.

Unlike Czarnecka et al. [85], our findings suggest that higher DLQI scores were associated with an increase in markers of systemic inflammation, such as leptin and IL-23, implying a link between BMI-driven inflammation and quality of life deterioration. This could indicate that in our cohort, the psychosocial burden of psoriasis as reflected by DLQI is directly influenced by systemic inflammatory pathways more than by BMI alone.

As we evaluated the connection between quality of life and systemic immune–inflammatory index (SII), we established a strong association. Notably, only 1.3% of the patients who were in DLQI class 3, indicative of a moderate quality of life, exhibited a higher SII risk, while the majority of the 8% in this subgroup had a low SII risk. At the same time, 26% of patients in DLQI class 5, which is characterized by a significant impairment in quality of life, were classified as having a high SII risk, indicating a strong correlation between amplified systemic inflammation and psychosocial burden. These results underline that as systemic inflammation increases, the quality of life deteriorates.

Similar to the findings of Gambichler et al. [86] and Cozma et al. [87], which emphasize the close links between SII, disease severity as measured by PASI score and the degree of psychoemotional impairment of life as measured by the DLQI score, our study uniquely categorizes DLQI classes and correlates them with specific SII risk levels. This approach provides deeper insight into how systemic inflammation may exacerbate not only clinical symptoms but also psychosocial challenges. Moreover, Hagino et al. [88] underlined in their study the potential of targeting systemic inflammation to improve patient outcomes, which is consistent with our study.

## 5. Conclusions

This study highlights the complex interaction between systemic inflammation, metabolic syndrome and quality of life in patients with psoriasis. While treatment for psoriasis have markedly reduced clinical severity, as reflected in the PASI scores, the DLQI score highlights a persistent impairment in quality of life, particularly in patients with MS. The disconnect between PASI improvements and DLQI scores underlines the profound psychosocial and systemic burden exerted by MS.

The main strength of this study lies in the in-depth analysis of inflammatory biomarkers, such as leptin, IL-23 and SII, alongside PASI and DLQI scores, providing a multidimensional view of the disease burden. By stratifying the results according to DLQI classes and correlating them with markers of systemic inflammation, this research offers new insights into the interplay between clinical severity, systemic comorbidities and psychosocial well-being.

However, it is important to consider the limitations of the study. Due to the retrospective design of this cross-sectional investigation, causal relationships cannot be definitively established. Subjectivity may be introduced by reliance on patient-reported outcomes, such as DLQI, which could potentially affect the assessment of quality of life. The complex interplay between PASI, DLQI and systemic inflammatory markers warrants further investigation in future longitudinal studies that will include a wide range of patient demographics.

## Figures and Tables

**Table 1:** Latest research on IL-17 and IL-23 therapies in psoriatic disease.

Title	Authors	Journal	Year
Therapeutics targeting the IL-23 and IL-17 pathway in psoriasis	Ghoreschi, Kamran, et al.	The Lancet	2021
Adverse events with IL-17 and IL-23 inhibitors for psoriasis and psoriatic arthritis: a systematic review and meta-analysis of phase III studies	N.D. Loft, et al.	Journal of the European Academy of Dermatology and Venereology	2020
Drug survival of IL-12/23, IL-17 and IL-23 inhibitors for psoriasis treatment: a retrospective multi-country, multicentric cohort study	Torres, T., et al.	American journal of clinical dermatology	2021
The TNF/IL-23/IL-17 axis—Head-to-head trials comparing different biologics in psoriasis treatment.	Ten Bergen, et al.	Scandinavian journal of immunology	2020
Efficacy and safety of risankizumab in psoriasis patients who failed anti-IL-17, anti-12/23 and/or anti IL-23: preliminary data of a real-life 16-week retrospective study.	Megna, M., et al.	Dermatologic Therapy	2020
Compositional alteration of gut microbiota in psoriasis treated with IL-23 and IL-17 inhibitors.	Huang, Y. H., et al.	International Journal of Molecular Sciences	2023
Human NCF190H Variant Promotes IL-23/IL-17—Dependent Mannan-Induced Psoriasis and Psoriatic Arthritis	Li, Y., et al.	Antioxidants	2023
Treatment of psoriasis patients with latent tuberculosis using IL-17 and IL-23 inhibitors: a retrospective, multinational, multicentre study	Torres, T., et al.	American Journal of Clinical Dermatology	2024
Biologic therapies targeting the interleukin IL-23/IL-17 immune axis for the treatment of moderate-to-severe plaque psoriasis: a systematic review and meta-analysis.	Erichsen, C. Y., et al.	Journal of the European Academy of Dermatology and Venereology	2020
Drug survival of interleukin IL-17 and IL-23 inhibitors for the treatment of psoriasis: a retrospective multi-country, multicentric cohort study.	Torres, T., et al.	American Journal of Clinical Dermatology	2022

**Table 2:** Main characteristics of the study subgroups.

Parameter	Values	TOTAL	PSO–MS	PSO	*p*-Value
		150 Patients	74 Patients	76 Patients	
Age Group (years old)	≤40	18 (12.0%)	2 (1.3%)	16 (10.7%)	0.007
	41–50	42 (28.0%)	22 (14.7%)	20 (13.3%)	
	51–60	40 (26.7%)	22 (14.7%)	18 (12.0%)	
	≥61	50 (33.3%)	28 (18.7%)	22 (14.7%)	
Gender	F	58 (38.7%)	20 (13.3%)	38 (25.3%)	<0.01
	M	92 (61.3%)	54 (36.0%)	38 (41.3%)	
BMI (kg/m^2^)	<18.5	0 (0%)	0 (0%)	0 (0%)	<0.01
	18.5–24.9	20 (13.3%)	4 (2.7%)	16 (10.7%)	
	25.0–29.9	46 (30.7%)	16 (10.7%)	30 (20%)	
	≥30.0	84 (56.0%)	54 (36%)	30 (20.0%)	
Comorbidities	Hypertrygliceridemia–BG (Y/N)	94 (62.7%)	74 (49.3%)	20 (13.3%)	<0.01
		56 (37.3%)	0 (0.0%)	56 (37.3%)	
	Hypertrygliceridemia–12MF (Y/N)	58 (38.7%)	50 (33.3%)	8 (5.3%)	<0.01
		92 (61.3%)	24 (16.0%)	68 (45.3%)	
	Diabetes mellitus (Y/N)	78 (52.0%)	62 (41.3%)	16 (10.7%)	<0.01
		72 (48.0%)	12 (8.0%)	60 (40.0%)	
	Low HDL-cholesterol–BG (Y/N)	66 (44.0%)	60 (40.0%)	6 (4.0%)	<0.01
		84 (56.0%)	14 (9.3%)	70 (46.7%)	
	Low HDL-cholesterol–12MF (Y/N)	6 (4.0%)	6 (4.0%)	68 (45.3%)	0.011
		144 (96.0%)	0 (0.0%)	76 (50.7%)	
	Arterial Hypertension–BG (Y/N)	78 (52.0%)	74 (49.3%)	4 (2.7%)	<0.01
		72 (48.0%)	0 (0.0%)	72 (48.0%)	
	Arterial Hypertension–12MF (Y/N)	78 (52.0%)	74 (49.3%)	4 (2.7%)	<0.01
		72 (48.0%)	0 (0.0%)	72 (48.0%)	
PASI Initial	mild psoriasis	0 (0%)	0 (0%)	0 (0%)	0.03
	moderate psoriasis	86 (57.3%)	36 (24.0%)	50 (33.3)	
	severe psoriasis	64 (42.7%)	38 (25.3%)	26 (17.3%)	
PASI 1 year	mild psoriasis	0 (0%)	0 (0%)	0 (0%)	<0.01
	moderate psoriasis	150 (100%)	74 (49.3%)	76 (50.7%)	
	severe psoriasis	0 (0%)	0 (0%)	0 (0%)	
DLQI Initial	0–1	0 (0%)	0 (0%)	0 (0%)	<0.01
	2–5	0 (0%)	0 (0%)	0 (0%)	
	6–10	14 (9.3%)	0 (0%)	14 (9.3%)	
	11–20	82 (54.7%)	34 (22.7%)	48 (32.0%)	
	21–30	54 (36.0%)	40 (26.7%)	14 (9.3%)	
DLQI 1 year	0–1	0 (0%)	0 (0%)	0 (0%)	<0.01
	2–5	0 (0%)	0 (0%)	0 (0%)	
	6–10	40 (26.7%)	36 (24.0%)	4 (2.7%)	
	11–20	68 (45.3%)	34 (22.7%)	34 (22.7%)	
	21–30	42 (28.0%)	4 (2.7%)	38 (25.3%)	
Cholesterol (mg/dL)	BG	231.8	238.1	225.7	<0.01
	12MF	206.8	208.9	204.8	
Triglycerides (mg/dL)	BG	209.1	281.8	138.3	<0.01
	12MF	143.5	180.9	107.2	
Glycaemia (mg/dL)		122.0	150.7	94.1	<0.01
Leptin (ng/mL)	BG	757.7	982.4	538.9	<0.01
	12MF	512.8	658	371.5	
IL-17A (pg/mL)		1064.7	1426.4	712.4	0.09
IL-23 (pg/mL)		694.5	903.7	490.8	0.02
HDL (mg/dL)	BG	49.2	39.1	59.1	<0.01
	12MF	56.7	51.9	61.4	

**Table 3:** Kendall’s tau-b correlation between all PASI score and clinical parameters.

Parameter	PASI	Tau-b Coefficient τb		*p*-Value ^1^
		PSO	PSO–MS	
IL-17A	3 months	0.08	0.08	<0.01
	6 months	0.11	−0.006	0.01
	12 months	0.09	0.005	0.01
IL-23	3 months	0.15	0.18	<0.01
	6 months	0.21	−0.07	0.01
	12 months	0.23	0.02	0.02
Leptin (initial)	3 months	−0.10	0.15	<0.01
	6 months	0.07	0.04	<0.01
	12 months	−0.01	0.28	<0.01
Leptin (12 months)	3 months	−0.004	0.37	0.01
	6 months	0.19	0.06	<0.01
	12 months	0.067	0.21	0.01

^1^ Kendall’s tau-b test.

**Table 4:** Analysis of the correlation among principal characteristics of the study subgroups.

Parameter	Tau-b Coefficient τb	*p*-Value ^1^
Age (years old)	0.07	0.21
Weight (kg)	0.19	0.01
AC (cm)	0.06	0.24
BMI	0.18	<0.01
Cholesterol	0.21	<0.01
Triglycerides	0.21	<0.01
Glycaemia	0.14	0.01
Leptin	0.17	0.01
IL-17A	−0.02	0.63
IL-23	0.14	0.01
HDL	−0.13	0.01

^1^ Mann–Whitney U test.

**Table 5:** Gender distribution to DLQI.

	Class	Gender	Frequency	Age			*p*-Value ^1^
			N (%)	Min	Max	Mean ± Stdev	
DLQI	3	F	4 (6.9)	41	52	46.5 ± 3.2	0.33
		M	10 (10.9)	37	65	53.2 ± 3.2	
	4	F	36 (62.1)	26	68	49.4 ± 1.7	
		M	46 (50)	26	76	56.2 ± 2.1	
	5	F	18 (31.0)	39	72	56.7 ± 2.5	
		M	36 (39.1)	35	74	55.3 ± 1.6	

^1^ Chi–square test.

**Table 6:** Correlation between key characteristics of the study subgroups.

Parameter	Tau-b Coefficient τb	*p*-Value ^1^
Age (years old)	0.08	0.18
Weight (kg)	0.16	0.01
CA (cm)	0.12	0.05
BMI	0.19	0.01
Cholesterol	0.22	0.01
Triglycerides	0.38	<0.01
Glycaemia	0.25	<0.01
Leptin	0.26	<0.01
IL-17A	0.11	0.07
IL-23	0.17	0.01
HDL	−0.20	0.01

^1^ Kendall’s tau-b test.

**Table 7:** Kruskal–Wallis test for DLQI score and PASI.

PASI	Median		*p*-Value ^1^
	PSO	PSO–MS	
0 months	18.46	22.3	<0.01
3 months	5.72	8.4	0.01
6 months	2.32	5.0	0.01
12 months	0.97	3.2	0.01

^1^ Kruskal–Wallis test.

**Table 8:** SII distribution to DLQI.

	Class	DLQI	SII		*p*-Value ^1^
		Number of Cases N (%)	Lower Critically Risk N (%)	Higher Critically Risk N (%)	
DLQI	3	14 (9.3%)	12 (8.0%)	2 (1.3%)	<0.01
	4	82 (54.7%)	40 (26.7%)	42 (28.0%)	
	5	54 (36.0%)	15 (10.0%)	39 (26.0%)	

## Data Availability

The authors declare that the data of this research are available from the corresponding authors upon reasonable request.

## References

[1] Horn E.J., Fox K.M., Patel V., Chiou C.-F., Dann F., Lebwohl M. (2007). Association of Patient-Reported Psoriasis Severity with Income and Employment. J. Am. Acad. Dermatol..

[2] Reich K. (2012). The Concept of Psoriasis as a Systemic Inflammation: Implications for Disease Management. J. Eur. Acad. Dermatol. Venereol..

[3] Elewski B., Alexis A.F., Lebwohl M., Stein Gold L., Pariser D., Del Rosso J., Yosipovitch G. (2019). Itch: An Under-Recognized Problem in Psoriasis. J. Eur. Acad. Dermatol. Venereol..

[4] Pithadia D.J., Reynolds K.A., Lee E.B., Wu J.J. (2019). Psoriasis-Associated Itch: Etiology, Assessment, Impact, and Management. J. Dermatol. Treat..

[5] Wintermann G., Bierling A.L., Peters E.M.J., Abraham S., Beissert S., Weidner K. (2024). Psychosocial Stress Affects the Change of Mental Distress under Dermatological Treatment—A Prospective Cohort Study in Patients with Psoriasis. Stress Health.

[6] Lebwohl M.G., Kavanaugh A., Armstrong A.W., Van Voorhees A.S. (2015). US Perspectives in the Management of Psoriasis and Psoriatic Arthritis: Patient and Physician Results from the Population-Based Multinational Assessment of Psoriasis and Psoriatic Arthritis (MAPP) Survey. Am. J. Clin. Dermatol..

[7] Takeshita J., Grewal S., Langan S.M., Mehta N.N., Ogdie A., Van Voorhees A.S., Gelfand J.M. (2017). Psoriasis and Comorbid Diseases. J. Am. Acad. Dermatol..

[8] Bu J., Ding R., Zhou L., Chen X., Shen E. (2022). Epidemiology of Psoriasis and Comorbid Diseases: A Narrative Review. Front. Immunol..

[9] Al-Mutairi N., Al-Farag S., Al-Mutairi A., Al-Shiltawy M. (2010). Comorbidities Associated with Psoriasis: An Experience from the Middle East. J. Dermatol..

[10] Gisondi P., Tessari G., Conti A., Piaserico S., Schianchi S., Peserico A., Giannetti A., Girolomoni G. (2007). Prevalence of metabolic syndrome in patients with psoriasis: A hospital-based case-control study. Br. J. Dermatol..

[11] Chen Y.-J., Wu C.-Y., Shen J.-L., Chu S.-Y., Chen C.-K., Chang Y.-T., Chen C.-M. (2008). Psoriasis Independently Associated With Hyperleptinemia Contributing to Metabolic Syndrome. Arch. Dermatol..

[12] Augustin M., Reich K., Glaeske G., Schaefer I., Radtke M. (2010). Co-morbidity and Age-related Prevalence of Psoriasis: Analysis of Health Insurance Data in Germany. Acta Derm.-Venereol..

[13] Parisi R., Symmons D.P.M., Griffiths C.E., Ashcroft D.M. (2013). Global Epidemiology of Psoriasis: A Systematic Review of Incidence and Prevalence. J. Investig. Dermatol..

[14] Souza C.S., de Castro C.C.S., Carneiro F.R.O., Pinto J.M.N., Fabricio L.H.Z., Azulay-Abulafia L., Romiti R., Cestari T.F., Suzuki C.E., Biegun P.M. (2018). Metabolic Syndrome and Psoriatic Arthritis among Patients with Psoriasis Vulgaris: Quality of Life and Prevalence. J. Dermatol..

[15] Gisondi P., Fostini A.C., Fossà I., Girolomoni G., Targher G. (2018). Psoriasis and the Metabolic Syndrome. Clin. Dermatol..

[16] Huang P.L. (2009). A Comprehensive Definition for Metabolic Syndrome. Dis. Model. Mech..

[17] Samburskaya O.V., Kalinchenko S.Y., Batkaeva N.V. (2021). BIOCHEMICAL PATHWAYS of METABOLIC DISORDERS in PSORIASIS. Juvenis Sci..

[18] Chan W., Yew Y., Theng C., Liew C., Oon H. (2020). Prevalence of Metabolic Syndrome in Patients with Psoriasis: A Cross-Sectional Study in Singapore. Singap. Med. J..

[19] Korman N.J. (2019). Management of Psoriasis as a Systemic Disease: What Is the Evidence?. Br. J. Dermatol..

[20] Patrick M.T., Stuart P.E., Zhang H., Zhao Q., Yin X., He K., Zhou X., Mehta N.N., Voorhees J.J., Boehnke M. (2021). Causal Relationship and Shared Genetic Loci between Psoriasis and Type 2 Diabetes through Trans-Disease Meta-Analysis. J. Investig. Dermatol..

[21] Gisondi P., Bellinato F., Girolomoni G., Albanesi C. (2020). Pathogenesis of Chronic Plaque Psoriasis and Its Intersection with Cardio-Metabolic Comorbidities. Front. Pharmacol..

[22] Mehta N.N., Teague H.L., Swindell W.R., Baumer Y., Ward N.L., Xing X., Baugous B., Johnston A., Joshi A.A., Silverman J. (2017). IFN-γ and TNF-α Synergism May Provide a Link between Psoriasis and Inflammatory Atherogenesis. Sci. Rep..

[23] Rose S., Stansky E., Dagur P.K., Samsel L., Weiner E., Jahanshad A., Doveikis J., Naik H.B., Playford M.P., Philip McCoy J. (2014). Characterization of Immune Cells in Psoriatic Adipose Tissue. J. Transl. Med..

[24] Gisondi P., Lora V., Bonauguri C., Russo A., Lippi G., Girolomoni G. (2013). Serum Chemerin Is Increased in Patients with Chronic Plaque Psoriasis and Normalizes Following Treatment with Infliximab. Br. J. Dermatol..

[25] Davidovici B.B., Sattar N., Jörg P.C., Puig L., Emery P., Barker J.N., van de Kerkhof P., Ståhle M., Nestle F.O., Girolomoni G. (2010). Psoriasis and Systemic Inflammatory Diseases: Potential Mechanistic Links between Skin Disease and Co-Morbid Conditions. J. Investig. Dermatol..

[26] Blackstone B., Patel R., Bewley A. (2022). Assessing and Improving Psychological Well-Being in Psoriasis: Considerations for the Clinician. Psoriasis Targets Ther..

[27] Huang T.-H., Lin C.-F., Alalaiwe A., Yang S.-C., Fang J.-Y. (2019). Apoptotic or Antiproliferative Activity of Natural Products against Keratinocytes for the Treatment of Psoriasis. Int. J. Mol. Sci..

[28] Scuderi S.A., Cucinotta L., Filippone A., Lanza M., Campolo M., Paterniti I., Esposito E. (2022). Effect of Melatonin on Psoriatic Phenotype in Human Reconstructed Skin Model. Biomedicines.

[29] Patel N., Nadkarni A., Cardwell L.A., Vera N., Frey C., Patel N., Feldman S.R. (2017). Psoriasis, Depression, and Inflammatory Overlap: A Review. Am. J. Clin. Dermatol..

[30] Pistorio M.L., Moretta T., Musumeci M.L., Russo C., Lacarrubba F., Petralia A., Micali G., Pasquale C.D. (2024). Impact of Attachment Style and Temperament Traits on the Quality of Life of Patients with Psoriasis. Behav. Sci..

[31] Dowlatshahi E.A., Wakkee M., Arends L.R., Nijsten T. (2014). The Prevalence and Odds of Depressive Symptoms and Clinical Depression in Psoriasis Patients: A Systematic Review and Meta-Analysis. J. Investig. Dermatol..

[32] Adesanya E.I., Matthewman J., Schonmann Y., Hayes J.F., Henderson A., Mathur R., Mulick A.R., Smith C.H., Langan S.M., Mansfield K.E. (2023). Factors Associated with Depression, Anxiety and Severe Mental Illness among Adults with Atopic Eczema or Psoriasis: A Systematic Review and Meta-Analysis. Br. J. Dermatol..

[33] Mazzotti E., Barbaranelli C., Picardi A., Abeni D., Pasquini P. (2005). Psychometric Properties of the Dermatology Life Quality Index (DLQI) in 900 Italian Patients with Psoriasis. Acta Derm.-Venereol..

[34] Finlay A.Y., Khan G.K. (1994). Dermatology Life Quality Index (DLQI)-a Simple Practical Measure for Routine Clinical Use. Clin. Exp. Dermatol..

[35] Lewis V., Finlay A.Y. (2004). 10 Years Experience of the Dermatology Life Quality Index (DLQI). J. Investig. Dermatol. Symp. Proc..

[36] Bulat V., Šitum M., Delaš Aždajić M., Lovrić I., Dediol I. (2020). Study on the Impact of Psoriasis on Quality of Life: Psychological, Social and Financial Implications. Psychiatr. Danub..

[37] Kimball A.B., Jacobson C., Weiss S., Vreeland M.G., Wu Y. (2005). The Psychosocial Burden of Psoriasis. Am. J. Clin. Dermatol..

[38] Ljosaa T.M., Bondevik H., Halvorsen J.A., Carr E., Wahl A.K. (2020). The Complex Experience of Psoriasis Related Skin Pain: A Qualitative Study. Scand. J. Pain.

[39] Mattei P.L., Corey K.C., Kimball A.B. (2013). Psoriasis Area Severity Index (PASI) and the Dermatology Life Quality Index (DLQI): The Correlation between Disease Severity and Psychological Burden in Patients Treated with Biological Therapies. J. Eur. Acad. Dermatol. Venereol..

[40] Houghton K., Patil D., Gomez B., Feldman S.R. (2021). Correlation between Change in Psoriasis Area and Severity Index and Dermatology Life Quality Index in Patients with Psoriasis: Pooled Analysis from Four Phase 3 Clinical Trials of Secukinumab. Dermatol. Ther..

[41] Mueller S.M., Itin P.H., Navarini A.A., Goldust M., Brandt O., Griffiths C.E.M., Kleyn C.E. (2020). The Relationship between PASI and DLQI with Itch, Stress, and Depression: Do We Need Additional Decision-Making Tools in Psoriasis?. Dermatol. Ther..

[42] Hu B., Yang X.-R., Xu Y., Sun Y.-F., Sun C., Guo W., Zhang X., Wang W.-M., Qiu S.-J., Zhou J. (2014). Systemic Immune-Inflammation Index Predicts Prognosis of Patients after Curative Resection for Hepatocellular Carcinoma. Clin. Cancer Res..

[43] Martínez-Ortega J.M., Nogueras P., Muñoz-Negro J.E., Gutiérrez-Rojas L., González-Domenech P., Gurpegui M. (2019). Quality of Life, Anxiety and Depressive Symptoms in Patients with Psoriasis: A Case-Control Study. J. Psychosom. Res..

[44] Fuat M.S.A., Yudin Z.M., Muhammad J., Zin F.M. (2022). Quality of Life and Its Associated Factors among Patients with Psoriasis in a Semi-Urban Northeast Malaysia. IJERPH.

[45] Devrimci-Ozguven H., Kundakci N., Kumbasar H., Boyvat A. (2000). The Depression, Anxiety, Life Satisfaction and Affective Expression Levels in Psoriasis Patients. J. Eur. Acad. Dermatol. Venereol..

[46] García-Sánchez L., Montiel-Jarquín Á.J., Vázquez-Cruz E., May-Salazar A., Gutiérrez-Gabriel I., Loría-Castellanoso J. (2017). Quality of life in patients with psoriasis. Gac. Medica Mex..

[47] Geale K., Henriksson M., Schmitt-Egenolf M. (2017). How is Disease Severity Associated with Quality of Life in Psoriasis Patients? Evidence from a Longitudinal Population-Based Study in Sweden. Health Qual. Life Outcomes.

[48] Malsawmtluangi C., Paul N., Panda S. (2023). Emotional Turbulence Perceived by Psoriasis Patients. Int. J. Sci. Healthc. Res..

[49] Golpour M., Hosseini S.H., Khademloo M., Ghasemi M., Ebadi A., Koohkan F., Shahmohammadi S. (2012). Depression and Anxiety Disorders among Patients with Psoriasis: A Hospital-Based Case-Control Study. Dermatol. Res. Pr..

[50] Zachariae R., Zachariae H., Blomqvist K., Davidsson S., Molin L., MOrk C., Sigurgeirsson B. (2002). Quality of Life in 6497 Nordic Patients with Psoriasis. Br. J. Dermatol..

[51] Fortune D.G., Richards H.L., Main C.J., Griffiths C.E.M. (1998). What Patients with Psoriasis Believe about Their Condition. J. Am. Acad. Dermatol..

[52] Tang J., Zhao S., Shi H., Li X., Ran L., Cao J., He Y. (2024). Effects on Peripheral and Central Nervous System of Key Inflammatory Intercellular Signalling Peptides and Proteins in Psoriasis. Exp. Dermatol..

[53] Riol-Blanco L., Ordovas-Montanes J., Perro M., Naval E., Thiriot A., Alvarez D., Paust S., Wood J.N., von Andrian U.H. (2014). Nociceptive Sensory Neurons Drive Interleukin-23-Mediated Psoriasiform Skin Inflammation. Nature.

[54] Marek-Jozefowicz L., Czajkowski R., Borkowska A., Nedoszytko B., Żmijewski M.A., Cubała W.J., Slominski A.T. (2022). The Brain–Skin Axis in Psoriasis—Psychological, Psychiatric, Hormonal, and Dermatological Aspects. Int. J. Mol. Sci..

[55] Hunter H.J.A., Griffiths C.E.M., Kleyn C.E. (2013). Does Psychosocial Stress Play a Role in the Exacerbation of Psoriasis?. Br. J. Dermatol..

[56] Hall J.M.F., des Anges C., Podawiltz A., Mummert D.I., Jones H., Mummert M.E. (2012). Psychological Stress and the Cutaneous Immune Response: Roles of the HPA Axis and the Sympathetic Nervous System in Atopic Dermatitis and Psoriasis. Dermatol. Res. Pract..

[57] Harvima I.T., Nilsson G. (2012). Stress, the Neuroendocrine System and Mast Cells: Current Understanding of Their Role in Psoriasis. Expert Rev. Clin. Immunol..

[58] Connor C.J., Liu V., Fiedorowicz J.G. (2015). Exploring the Physiological Link between Psoriasis and Mood Disorders. Dermatol. Res. Pr..

[59] Reddy P., Lent-Schochet D., Ramakrishnan N., McLaughlin M., Jialal I. (2019). Metabolic Syndrome Is an Inflammatory Disorder: A Conspiracy between Adipose Tissue and Phagocytes. Clin. Chim. Acta.

[60] Elks C.M., Francis J. (2010). Central Adiposity, Systemic Inflammation, and the Metabolic Syndrome. Curr. Hypertens. Rep..

[61] Xu T., Zhang Y.-H. (2012). Association of Psoriasis with Stroke and Myocardial Infarction: Meta-Analysis of Cohort Studies. Br. J. Dermatol..

[62] Masson W., Lobo M., Molinero G. (2020). Psoriasis and Cardiovascular Risk: A Comprehensive Review. Adv. Ther..

[63] Mosca M., Hong J., Hadeler E., Hakimi M., Brownstone N., Liao W., Bhutani T. (2021). Psoriasis and Cardiometabolic Comorbidities: An Evaluation of the Impact of Systemic Treatments in Randomized Clinical Trials. Dermatol. Ther..

[64] Ryan C., Kirby B. (2015). Psoriasis is a Systemic Disease with Multiple Cardiovascular and Metabolic Comorbidities. Dermatol. Clin..

[65] Wu J.J., Kavanaugh A., Lebwohl M.G., Gniadecki R., Merola J.F. (2022). Psoriasis and Metabolic Syndrome: Implications for the Management and Treatment of Psoriasis. J. Eur. Acad. Dermatol. Venereol..

[66] Hu S., Lan C.-C.E. (2017). Psoriasis and Cardiovascular Comorbidities: Focusing on Severe Vascular Events, Cardiovascular Risk Factors and Implications for Treatment. Int. J. Mol. Sci..

[67] Hao Y., Zhu Y., Zou S., Zhou P., Hu Y., Zhao Q., Gu L., Zhang H., Wang Z., Li J. (2021). Metabolic Syndrome and Psoriasis: Mechanisms and Future Directions. Front. Immunol..

[68] Anyfanti P., Margouta A., Goulas K., Gavriilaki M., Lazaridou E., Patsatsi A., Gkaliagkousi E. (2022). Endothelial Dysfunction in Psoriasis: An Updated Review. Front. Med..

[69] Ito F., Sono Y., Ito T. (2019). Measurement and Clinical Significance of Lipid Peroxidation as a Biomarker of Oxidative Stress: Oxidative Stress in Diabetes, Atherosclerosis, and Chronic Inflammation. Antioxidants.

[70] Kang H., Li X., Xiong K., Song Z., Tian J., Wen Y., Sun A., Deng X. (2021). The Entry and Egress of Monocytes in Atherosclerosis: A Biochemical and Biomechanical Driven Process. Cardiovasc. Ther..

[71] Langan S.M., Seminara N.M., Shin D.B., Troxel A.B., Kimmel S.E., Mehta N.N., Margolis D.J., Gelfand J.M. (2012). Prevalence of Metabolic Syndrome in Patients with Psoriasis: A Population-Based Study in the United Kingdom. J. Investig. Dermatol..

[72] Liakou A., Zouboulis C. (2015). Links and Risks Associated with Psoriasis and Metabolic Syndrome. Psoriasis Targets Ther..

[73] Orloff J.N., Kaminetsky J.R., Aziz M. (2018). Psoriasis and Obesity: A Review of the Current Literature. Ski. J. Cutan. Med..

[74] Nakhwa Y.C., Rashmi R., Basavaraj K.H. (2014). Dyslipidemia in Psoriasis: A Case Controlled Study. Int. Sch. Res. Not..

[75] Gisondi P., Del Giglio M., Girolomoni G. (2016). Considerations for Systemic Treatment of Psoriasis in Obese Patients. Am. J. Clin. Dermatol..

[76] Merola J.F., Kavanaugh A., Lebwohl M.G., Gniadecki R., Wu J.J. (2022). Clinical Efficacy and Safety of Psoriasis Treatments in Patients with Concomitant Metabolic Syndrome: A Narrative Review. Dermatol. Ther..

[77] Gargiulo L., Ibba L., Malagoli P., Amoruso F., Argenziano G., Balato A., Bardazzi F., Burlando M., Carrera C.G., Damiani G. (2023). Brodalumab for the Treatment of Plaque Psoriasis in a Real-Life Setting: A 3 Years Multicenter Retrospective Study—IL PSO (Italian Landscape Psoriasis). Front. Med..

[78] Ibba L., Di Giulio S., Gargiulo L., Facheris P., Perugini C., Costanzo A., Narcisi A., Valenti M. (2024). Long-Term Effectiveness and Safety of Risankizumab in Patients with Moderate-To-Severe Psoriasis with and without Cardiometabolic Comorbidities: A Single-Center Retrospective Study. J. Dermatol. Treat..

[79] Yang J., Hu K., Li X., Hu J., Tan M., Zhang M., Chen J., Kuang Y. (2023). Psoriatic Foot Involvement Is the Most Significant Contributor to the Inconsistency Between PASI and DLQI: A Retrospective Study from China. CCID.

[80] Räikkönen K., Matthews K.A., Kuller L.H. (2007). Depressive Symptoms and Stressful Life Events Predict Metabolic Syndrome Among Middle-Aged Women. Diabetes Care.

[81] Katano S., Nakamura Y., Nakamura A., Suzukamo Y., Murakami Y., Tanaka T., Okayama A., Miura K., Okamura T., Fukuhara S. (2012). Relationship between Health-Related Quality of Life and Clustering of Metabolic Syndrome Diagnostic Components. Qual. Life Res..

[82] Ford E.S., Li C. (2008). Metabolic Syndrome and Health-Related Quality of Life among U.S. Adults. Ann. Epidemiol..

[83] Lin Y.-H., Chang H.-T., Tseng Y.-H., Chen H.-S., Chiang S.-C., Chen T.-J., Hwang S.-J. (2021). Changes in Metabolic Syndrome Affect the Health-Related Quality of Life of Community-Dwelling Adults. Sci. Rep..

[84] Pearl R.L., Wadden T.A., Hopkins C.M., Shaw J.A., Hayes M.R., Bakizada Z.M., Alfaris N., Chao A.M., Pinkasavage E., Berkowitz R.I. (2017). Association between Weight Bias Internalization and Metabolic Syndrome among Treatment-Seeking Individuals with Obesity. Obesity.

[85] Czarnecka A., Zabłotna M., Purzycka-Bohdan D., Nowicki R.J., Szczerkowska-Dobosz A. (2023). An Observational Study of 147 Psoriasis Patients: Overweightness and Obesity as a Significant Clinical Factors Correlated with Psoriasis. Medicina.

[86] Gambichler T., Würfel L., Abu Rached N., Mansour R., Bechara F.G., Scheel C.H. (2023). Systemic Immune-inflammation Biomarkers in Psoriasis Patients under Interleukin 17A -inhibitor Treatment. Acad. Dermatol. Venereol..

[87] Cozma E.C., Găman M.-A., Orzan O., Hamed K.-V., Voiculescu V.M., Găman A.-M. (2023). Oxidative Stress and Inflammation Levels in a Population of Eastern European Naïve Versus Treated Psoriasis Patients. Cureus.

[88] Hagino T., Saeki H., Fujimoto E., Kanda N. (2024). Long-Term Effectiveness and Safety of Deucravacitinib in Psoriasis: A 52-Week Real-World Study of Genital, Scalp, and Nail Lesions. Clin. Exp. Dermatol..

